# Method for the Intraoperative Detection of IDH Mutation in Gliomas with Differential Mobility Spectrometry

**DOI:** 10.3390/curroncol29050265

**Published:** 2022-05-04

**Authors:** Ilkka Haapala, Anton Kondratev, Antti Roine, Meri Mäkelä, Anton Kontunen, Markus Karjalainen, Aki Laakso, Päivi Koroknay-Pál, Kristiina Nordfors, Hannu Haapasalo, Niku Oksala, Antti Vehkaoja, Joonas Haapasalo

**Affiliations:** 1 Department of Neurosurgery, Tampere University Hospital, 33520 Tampere, Finland; joonas.haapasalo@gmail.com; 2 Faculty of Medicine and Health Technology, Tampere University, 33014 Tampere, Finland; anton.kondratev@tuni.fi (A.K.); antti.roine@olfactomics.fi (A.R.); meri.makela@olfactomics.fi (M.M.); anton.kontunen@tuni.fi (A.K.); markus.karjalainen@tuni.fi (M.K.); niku.oksala@olfactomics.fi (N.O.); antti.vehkaoja@tuni.fi (A.V.); 3 Olfactomics Ltd., 33720 Tampere, Finland; 4 Department of Neurosurgery, Helsinki University Hospital, 00260 Helsinki, Finland; aki.laakso@hus.fi (A.L.); paivi.koroknay-pal@hus.fi (P.K.-P.); 5 Faculty of Pediatrics, Tampere University Hospital, 33520 Tampere, Finland; kristiina.nordfors@gmail.com; 6 Fimlab Laboratories Ltd., 33520 Tampere, Finland; hannu.haapasalo@fimlab.fi

**Keywords:** differential mobility spectrometry, neuro-oncology, neurosurgery, glioma, classification, isocitrate dehydrogenase (IDH)

## Abstract

Isocitrate dehydrogenase (IDH) mutation status is an important factor for surgical decision-making: patients with IDH-mutated tumors are more likely to have a good long-term prognosis, and thus favor aggressive resection with more survival benefit to gain. Patients with IDH wild-type tumors have generally poorer prognosis and, therefore, conservative resection to avoid neurological deficit is favored. Current histopathological analysis with frozen sections is unable to identify IDH mutation status intraoperatively, and more advanced methods are therefore needed. We examined a novel method suitable for intraoperative IDH mutation identification that is based on the differential mobility spectrometry (DMS) analysis of the tumor. We prospectively obtained tumor samples from 22 patients, including 11 IDH-mutated and 11 IDH wild-type tumors. The tumors were cut in 88 smaller specimens that were analyzed with DMS. With a linear discriminant analysis (LDA) algorithm, the DMS was able to classify tumor samples with 86% classification accuracy, 86% sensitivity, and 85% specificity. Our results show that DMS is able to differentiate IDH-mutated and IDH wild-type tumors with good accuracy in a setting suitable for intraoperative use, which makes it a promising novel solution for neurosurgical practice.

## 1. Introduction

Gliomas represent the most clinically important group of primary brain tumors. Traditionally, they have been classified into WHO groups I—IV to evaluate their malignant potential by analysis of their morphological features. However, the past decades of research have led to the discovery of many molecular alterations in gliomas that have a great impact on the tumor’s malignancy and, accordingly, to the patient’s prognosis [1]. Among such alterations, the mutation of isocitrate dehydrogenase (IDH) enzymes 1 or 2 is highly correlated with the patient’s overall survival, and the effect is present regardless of the tumor’s histopathological WHO grade [2,3,4,5]. IDH mutation also seems to play a pivotal role in the carcinogenesis of other solid tumors, such as cholangiocarcinoma, where it is also a major target for medical therapy [6,7,8].

Normally, IDH enzymes catalyze the oxidative decarboxylation of isocitrate to form a-ketoglutarate (aKG) in the Krebs cycle. IDH1 and IDH2 localize differently in the cell but share the same function; hence, they are hereafter referred to collectively as IDH. The mutation of IDH confers a neomorphic enzyme activity that catalyzes the reduction of aKG into the putative oncometabolite D-2-hydroxyglutarate (D2HG) [9]. The accumulation of D2HG is further associated with the hypermethylation of DNA and chromatin, which is thought to dysregulate cell epigenetics [10,11].

IDH mutation status is an important factor for surgical decision-making: patients with IDH-mutated tumors are more likely to have a good long-term prognosis, and thus favor aggressive gross total resection with more survival benefit to gain. Patients with IDH wild-type tumors have a generally poorer prognosis and, therefore, conservative resection to avoid neurological deficit is favored [12,13,14]. The effect of gross total resection on survival remains also in recurrent diseases [15,16]. Current histopathological analysis based on frozen sections is unable to identify molecular characteristics, including IDH mutation, within the time frame of surgery [17], thus creating an imminent need for new solutions.

We have previously shown that differential mobility spectrometry (DMS) is able to identify different brain tumors ex vivo [18]. DMS characterizes substances based on the mobility differences of ionized particles in high-frequency electrical fields, resulting in a substance-specific dispersion spectrum, or “smell fingerprint” [19]. The simplicity, quickness and cost-effectiveness of DMS makes it a compelling emerging technology for clinical applications [18]. In this study, we demonstrate the rapid, preparation-free analysis of a tumor’s IDH mutation status with DMS.

## 2. Materials and Methods

We prospectively obtained tumor samples from 22 patients who had neurosurgical operations at Tampere University Hospital between the years 2018 and 2021, and at Helsinki University Hospital in 2020. Patient recruitment was continued until we had a sufficient number of IDH-mutated tumors, which are rarer. To make balanced classes, an equal number of IDH wild-type tumors were randomly selected for the experiment. Eventually, we had 11 IDH-mutated tumors and 11 IDH wild-type tumors. IDH-mutated tumors included 5 WHO gr. II–III astrocytomas, 3 gr. II–III oligodendrogliomas, and 3 gr. IV glioblastomas (GBM). IDH wild-type tumors included 1 gr. III astrocytoma and 10 GBMs. Diagnoses were made by an experienced neuropathologist and IDH mutation was identified with immunohistochemistry. The study was approved by the ethics review board of Pirkanmaa Hospital District, Finland. The patients gave their written consent for the study.

**Figure 1:**
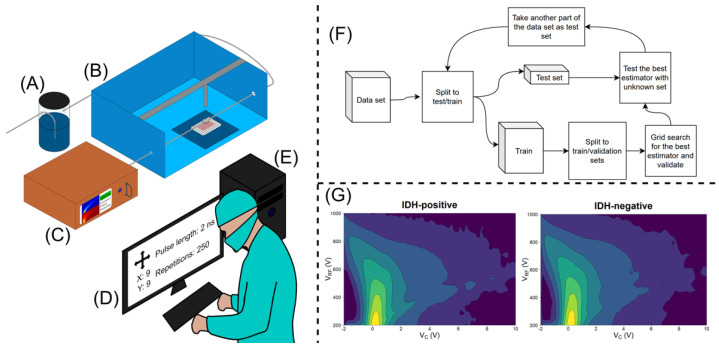
The setup: (**A**) humidifier; (**B**) sampling unit; (**C**) DMS analyzer (**D**); graphical user interface; (**E**) computing unit for data analytics; (**F**) workflow of the algorithm; (**G**) examples of IDH−positive and −negative dispersion spectra. Vc = compensation voltage; Vrf = peak-to-peak amplitude of the radiofrequency waveform voltage.

All samples were stored in a freezer at −70 °C. The samples were carefully cut into 88 (44 IDH-mutated and 44 IDH wild-type) smaller specimens of macroscopically equal sizes. Blood, if macroscopically visible, was carefully rinsed from the samples before the analysis. The samples were randomly placed in a plastic well plate with each well containing 0.18 mL of agar in the bottom. Each sample was incised with a custom-built, computer-controlled, 40 W, 10.6 μm CO_2_ laser evaporator four times in a quadratic manner, with 1 mm gaps between the incisions. The total number of incisions was 352. The laser sampling was controlled by a graphical user interface. To provide a clean and controlled supply of carrier gas for the analyte gas, purified and humidified pressurized air was introduced to the sampling stage via a sampling nozzle. The sampling nozzle provided a protective stream of carrier gas around the sampling area and, after sample vaporization, transported the sample gas to the DMS inlet. The DMS used in the study was a commercial IonVision instrument (Olfactomics Oy, Finland). The measurement parameters for the DMS spectrum were: separation voltage (Usv), 200–1000 V with 20 increments; compensation voltage (Ucv), −2–10 V with 60 increments; separation field frequency, 1 MHz; and duty cycle of the field, 22%. With these parameters, the DMS measurement produced a total of 1200 data points and the duration of the measurement was approximately 13 s, during which 250 2 ms laser pulses were used to provide a sample stream of vaporized tissue to the DMS. A gross appearance of the setup (A–D) and examples of the dispersion spectra (G) are presented in Figure 1.

We evaluated the accuracy of several machine learning algorithms for the detection of differences in dispersion spectra and the classification of the analyzed samples. Linear discriminant analysis (LDA) was found to be the best performing algorithm. The main idea of training an LDA algorithm is the projection of data points to a lower dimensional space so that the between-class distance of class centers is maximized, and the within-class distance of data points is minimized, defining a decision boundary between the classes that is used to classify new samples. The other algorithms tested were K-nearest neighbors (KNN), random forest (RF), decision tree (DT), support vector machines (SVM) and XGBoost (XGB).

## 3. Results

The data set revealed a temperature rise, which caused baseline drift during the measurement of one well plate, making the data biased. Thus, a necessary preprocessing method was to remove the dimension-wise linear trend which belonged the well plate from each part of the data set. This preprocessing step improved the classification results compared to the classification of the raw data. The data set contained 352 samples taken from 22 patients. Group cross-validation was utilised to estimate the classification performance. Group cross-validation is implemented so that, at every iteration, it leaves one group of samples only for testing. The other groups are used for training. In this case, the nested group cross-validation technique was used. This algorithm leaves one group for testing and the other groups are used for training and validating. For the next iteration, the second group is used for testing and the others for training and validating, and so on. This approach ensures that there are no data leakages into the training phase. With the nested group cross-validation training, the LDA algorithm reached a classification accuracy of 86%, with 86% sensitivity and 85% specificity (Table 1). The workflow of the LDA algorithm is presented in Figure 1F. Further details of the cross-validation and classification results reached with other algorithms are presented in the Supplementary File.

In terms of the samples, out of the original 22 tumor samples (352 incisions), 8 samples had all their incisions correctly classified. In five samples, less than 10% of incisions were erroneous. In four samples, 10–20% were wrong. In five samples, 20–50% of the incisions were incorrectly classified. The tumors that had incorrectly clustered incisions included eight IDH wild-type tumors and six IDH-mutated tumors. The most difficult tumor type for the classifier was gr. IV GBM.

## 4. Discussion

Our results show that the smoke generated from the IDH-mutated and IDH wild-type gliomas had distinct DMS profiles, and the DMS could differentiate them with good sensitivity and specificity. The laser evaporator platform is compact enough to be placed in the operating room and used for intermittent analysis of the tumor samples during surgery. The duration of measurement was approximately 13 s, so the DMS operates in almost real time. The DMS is also simpler and more economical than conventional mass spectrometer-based solutions. Conventional frozen section analysis is unable to identify molecular alterations in tumors, such as IDH mutation. In the latest WHO tumor classification, these alterations have become ever more prominent. This creates an increasing need for novel tumor identification methods in neurosurgical departments worldwide.

Recently, Raman spectroscopy has also been used for genotyping unprocessed glioma samples [20]. Raman spectroscopy is a modality that gives spectral tissue characteristics based on molecular signatures resulting from the inelastic scattering of incident light. Our results equal those achieved with Raman spectroscopy, and the workflow in DMS is at least as fast and straightforward.

Our tumor sample set included both IDH-mutated and IDH wild-type gr. IV GBMs and gr. III malignant astrocytomas. Out of the tumors with an unusual IDH mutation status given their histology, one GBM had 25% (9 out of 36) of the incisions erroneously classified, but all the other tumors (two IDH-mutated gr. IV GBMs and one IDH wild-type gr. III astrocytoma) had all their incisions correct classified, even though the opposite cluster had multiple histologically similar tumors. This indirectly indicates that the divisive features in the classification process were actually due to the cellular metabolic changes driven by an IDH mutation. The phospholipid content of tissue has previously been identified as a key distinguishing factor in DMS analysis [18]. The metabolic changes associated with an IDH mutation include aberrations in phospholipid composition [10], which constitutes a plausible theoretical basis for the detection of IDH mutation by DMS.

A potential source of error in DMS analysis is intratumoral heterogeneity. This is especially true in GBMs, which vary in terms of cellular density, nuclear pleomorphism, necrosis, histologic architecture, vasculature, mitoses, and multifaceted microenvironments [21,22]. This can cause variance in tissue impedance and disturb the classifier [23]. An additional confounding factor in our study was 5-ALA, which was used only in the resections of tumors that radiologically appeared as malignant. However, all three IDH-mutated GBMs were resected with 5-ALA guidance, and still the classifier was able to classify them correctly.

Our study was limited by a relatively small number of samples that we multiplied into smaller specimens. In order to achieve a setup resembling actual intraoperative use, we only minimally prepared the tumor samples for the analysis. This inevitably caused spatial variance in the specimens that affected the DMS signal strength, thus creating an additional confounding factor to the classifier. This issue could be addressed in future studies by processing the samples into a more homogeneous cell suspension by a centrifuge before the analysis. The suspension could then be pipetted into the well plate to obtain precisely equal sample sizes. We also used frozen samples instead of fresh tumors. In our earlier unpublished experiments, freezing of the samples was not found to affect the classification results. However, this should be verified in peer-reviewed studies in the future.

## 5. Conclusions

Our results show that the DMS is able to differentiate IDH-mutated and IDH wild-type tumors with good accuracy in a setting suitable for intraoperative use. The role of molecular alterations in classifying brain tumors and evaluating their prognosis is increasing. Additionally, the degree of survival benefit achieved with a gross-total resection varies even in histologically similar tumors based on their IDH mutation status, which is impossible to identify with conventional frozen section analysis. This makes the DMS a promising novel tool for neurosurgical practice.

## Figures and Tables

**Table 1:** Cross tabulation of the classification results (LDA).

IDH Mutation	−	150	26
	+	25	151
		−	+
		Classification result	
Sens. 0.85		Spec. 0.85	

## Data Availability

The data presented in this study are available on request from the corresponding author.

## Supplementary Materials

The following supporting information can be downloaded at: https://www.mdpi.com/article/10.3390/curroncol29050265/s1. The work includes a supplementary file; detailed description of data analysis and classification results achieved with other algorithms. Figure S1: Nested cross-validation.
