# Supplementary material for: Method for the Intraoperative Detection of IDH Mutation in Gliomas with Differential Mobility Spectrometry
Source: Curr Oncol. 2022 May 4;29(5):3252–8. doi: 10.3390/curroncol29050265 (PMC9139325; doi:10.3390/curroncol29050265)
Supplement: Supplementary file 1 [file curroncol-29-00265-s001.zip › curroncol-1589436-supplementary.pdf]

Current Oncology

MDPI

# Supplementary material for Method for the Intraoperative Detection of IDH Mutation in Gliomas with Differential Mobility Spectrometry

**Data analysis**

This section describes the machine learning algorithms evaluated for the differentiation between mutated and non-mutated tumors. The algorithms were: Linear Discriminant Analysis (LDA), K-Nearest Neighbors (KNN), Decision Tree (DT), Random Forest (RF), Support Vector Machines (SVM) and XGBoost (XGB).

The LDA algorithm is a well-known method for dimensionality reduction and classification. The main idea of training an LDA algorithm is the projection of data points to a lower dimensional space so that the between-class distance of class centers is maximized and the within-class distance of data points is minimized, and defining a decision boundary between the classes that is used to classify new samples. The KNN algorithm is a very popular, powerful and simple algorithm. It takes a sample, finds the closest K-Neighboring points in the training data and makes a prediction based on the majority vote. The DT algorithm generates optimized tree structure of the data and makes predictions by following the generated tree. The RF algorithm is an extension of the DT, where classification is based on several generated decision trees. The SVM algorithm in its simplest case tries to separate a data set by finding the smallest margin between a decision boundary and the closest samples to the decision boundary. The XGBoost algorithm is very close to the Random Forest concept but uses another algorithm for training.

The data set revealed temperature rise, which caused baseline drift during measurement of one well plate making the data biased. Thus, the only preprocessing method was removing dimension-wise linear trend from each part of the data set, which belongs to one well plate. This preprocessing step improved the classification results compared to the classification of the raw data. The data set contained 352 samples taken from 22 patients. Such structure of the data suggests using group cross-validation strategies for training algorithms. The group cross-validation is implemented such that at every iteration it leaves one group of samples only for testing. Other groups are used for training. In this case the nested group cross-validation technique was used. This algorithm leaves one group for testing and the rest groups are used for training and validating. For the next iteration the next group is used for testing and the rest for the training and validating and so on. This approach ensures that there are no data leakages into the training phase. Figure 2 shows visualization of the nested group cross-validation approach.

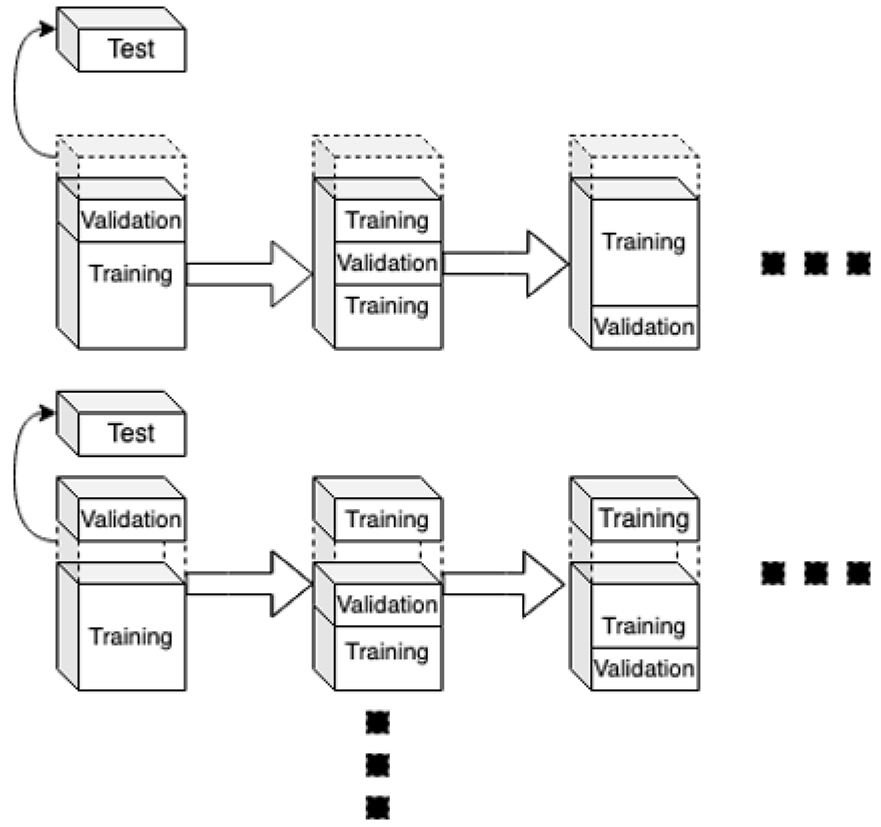


**Figure S1.** Nested cross-validation.

**Cross-validation. Details.**

The grid search is a popular technique to select the best parameters for a classifier. The grid search goes through all combinations of a predefined parameter set for determining the best combination of parameters. In this work the grid search was used inside the nested group cross-validation for exploring general classification capability of each applied algorithm. As can be seen from Figure 1 (Workflow of the algorithm), at the start the data is divided into training and test sets. The test set contains one group of patients and the train set contains the rest of the groups. The train set is divided into the train and validation sets. The latter two sets are used for the grid search and validation. When the best estimator is selected its accuracy is measured by predicting labels of the test set. On the next iteration the algorithm takes another group as the test and proceeds. The algorithm iterates over all 22 groups of patients. At each iteration the best model predicted the test set. The predictions and ground truths were stored in a separate vector for further analysis of accuracy.

**Set of parameters for the grid search**

See below for parameters used for each algorithm for training. The parameter grid used for each algorithm was:

| | | | |
|---|---|---|---|
| - | Linear Discriminant Analysis | - | K-Nearest Neighbors |
| - | solver: svd, lsqr | - | n_neighbors: 5,7,9,...,21 |
| - | store_covariance: True, False | - | weights: uniform, distance |
| - | shrinkage: None, 0.1,0.2,...,0.9, auto | - | algorithm: auto, ball_tree, kd_tree, brute |
| | | - | p: 1,2 |
| - | XGBoost | - | Decision Tree |
| - | n_estimators: 50, 100, 150, 200 | - | criterion: gini, entropy |

| | | | |
|---|---|---|---|
| - | learning_rate: 0.01, 0.1, 0.2, 0.3 | - | splitter: best, random |
| - | max_depth: 3,4,...,10 | - | max_features: auto, sqrt, log2, None |
| - | colsample_by_tree: 1/10, 2/10, 3/10 | | |
| - | gamma: 0, 1/10, 2/10 | | |
| - | eval_metric: error | | |
| - | Random Forest | - | Support Vector Machine |
| - | n_estimators: 10,20,30,...,90,100,150,200 | - | kernel: rbf, linear, poly, sigmoid |
| - | criterion: gini, entropy | - | gamma: scale, auto |
| - | max_features: auto, sqrt, log2 | - | shrinking: True, False |
| - | bootstrap: True, False | - | degree: 1,2,3,4 |
| - | warm_start: True, False | | |

The parameter's names are named as they are in the scikit-learn library. For a more detailed explanation on each parameter please refer to the scikit-learn official documentation.

**Classification results for other algorithms**

| | LDA | DT | RF | SVM | KNN | XGBoost |
|---|---|---|---|---|---|---|
| sensitivity | 0.86 | 0.60 | 0.71 | 0.79 | 0.61 | 0.74 |
| specificity | 0.85 | 0.63 | 0.72 | 0.78 | 0.57 | 0.74 |
| accuracy | 0.86 | 0.59 | 0.70 | 0.80 | 0.62 | 0.74 |

Classification results of different algorithms.
